# Approaches to Inducing β-Cell Regeneration

**DOI:** 10.3390/biomedicines10030571

**Published:** 2022-02-28

**Authors:** Fred Levine

**Affiliations:** SBP Medical Discovery Institute, La Jolla, CA 92037, USA; flevine@sbpdiscovery.org

**Keywords:** β-cell, islet, diabetes, regeneration, stem cell, pancreas, insulin

## Abstract

β-cell number and/or function is reduced in diabetes. Thus, inducing the formation of new β-cells has been a major goal of diabetes research. However, the pathway(s) by which new β-cells form when preexisting β-cells are decreased in number or cease to function has remained obscure. Many pathways have been proposed, but definitive evidence, particularly in humans, has been lacking. Replication of preexisting β-cells, neogenesis from ducts, redifferentiation from β-cells that dedifferentiated under metabolic stress, and transdifferentiation from other cell types, particularly within the islet, are the major mechanisms that have been proposed for generating increased numbers of functional β-cells. Here, I will discuss those approaches critically, with particular attention to transdifferentiation of preexisting α-cells to β-cells.

## 1. Introduction

That β-cell function and β-cells themselves are decreased in both major forms of diabetes has been known for many decades [1]. Strikingly, there continues to be controversy about the mechanism by which β-cell function is compromised. Questions, as fundamental as whether diabetes (either type 1 or 2) leads to β-cell death or merely to loss of markers of β-cell identity, whether β-cell replication can occur in adults with diabetes, and whether β-cell neogenesis from precursors is possible, remain without definitive answers. This review will discuss the controversies that exist in the field of restoration of functional β-cells, focusing on approaches amenable to pharmacological intervention and that act on pathways that are physiologically relevant to biologically active pathways that maintain β-cell mass under homeostatic or pathological conditions.

## 2. β-Cell Replication

It is clear that β-cell replication occurs early during development [2]. However, it is also clear that β-cell replication declines to undetectable levels in adults, and particularly in adult humans [3,4,5]. Thus, inducing β-cell replication in adults depends on reactivating a process that does not normally occur and that may be impossible due to β-cell alterations that are irreversible. Regardless, there have been many attempts to induce adult β-cell replication, most prominently by the use of inhibitors of the protein kinase Dyrk [6]. However, evidence of a substantial increase in the number of β-cells by stimulation of β-cell replication in adults remains lacking.

An important issue with studies of β-cell replication is the endpoint used to define that a β-cell has replicated. While the gold standard should be to demonstrate an increase in the number of β-cells, that has proven to be elusive. Most studies have used surrogate markers to indicate DNA replication. The most common are uptake of nucleoside analogs, such as BrdU or EdU, and expression of proteins, such as Ki67, that are expressed only in cells that have entered the cell cycle. However, nucleoside analog uptake, as an indicator of replication, can also be an indication of a DNA damage response, where repair involves incorporation of nucleosides [7]. The pattern of nucleoside uptake can indicate which is taking place, with an irregular, incomplete uptake in the nucleus being more consistent with a damage response, while a strong, uniform uptake being more consistent with true replication [8]. The presence of doublets of such cells further supports true replication, as the formation of new cells by replication will result in two daughter cells in close proximity. The expression of H2aX, a marker of a DNA damage response, has been found in some studies, including in some where β-cell replication has been claimed [7]. Ki67 expression can also be problematic as a marker of replication, as it has become clear that it plays a broader role in the cell [9]. In summary, studies that claim to show beta cell replication should demonstrate appropriate expression of markers, such as CENPA (Centromere Protein A) that are required for replication and the absence of markers of DNA damage that can produce false positive results in replication assays. Ultimately, strong evidence of an increase in β-cell number needs to be shown.

## 3. β-Cell Neogenesis from Ducts

Embryonic islet formation occurs by budding from embryonic ducts [10]. This gives rise to the hypothesis that β-cell regeneration in the adult occurs by reactivation of the embryonic program of islet development [11]. There is considerable evidence for the reactivation of embryonic programs in a number of tissues [12] as well as in cancer. Thus, it is a highly attractive notion and has dominated the field of β-cell regeneration for much of its history. Evidence to support β-cell regeneration from ducts through reactivation of the embryonic program of islet development has been tested primarily by the use of models in which various types of pancreatic injury is induced, followed by monitoring to detect islet or β-cell neogenesis. Many investigators, including us, have observed cells expressing insulin within ducts [13,14,15]. A transgenic mouse model expressing interferon-gamma in β-cells was claimed to lead to massive formation of insulin-positive cells within ducts [16], but there were no follow up studies from other laboratories to demonstrate reproducibility. While multiple models have been used to study the appearance and fate of intra-ductal insulin-positive cells, including partial pancreatectomy, duct ligation, pregnancy, and secreted factors from the fetal pancreas and other states—discussed below—substantial controversy remains about whether pancreatic ductal cells in adult mammals give rise to mature β-cells.

### 3.1. Partial Pancreatectomy

The classical model used to induce pancreatic damage has been a partial pancreatectomy [17]. In fact, the origin of the hypothesis that β-cell regeneration occurred by reactivation of an embryonic program came from a study of partial pancreatectomy [11]. That study demonstrated regenerating ducts following a partial pancreatectomy that had a high degree of BrdU incorporation, indicating that they were derived from replicating ductal precursors. An adjacent islet was claimed to be neogenic, with a ductal origin, but few of the islet cells exhibited BrdU incorporation, indicating that they had not arisen from a replicating precursor. The presence of ducts in which most cells had replication was taken as evidence of pancreatic regeneration and so the presence of an islet in that region was thought to indicate islet neogenesis. However, the islet shown had few cells that had taken up BrdU, indicating that most cells in that islet were preexisting rather than new.

Despite many publications on islet neogenesis following partial pancreatectomy, definitive evidence was lacking. If islet neogenesis occurred following partical pancreatectomy, new islets marked by many BrdU-positive endocrine cells should be found in neogenic areas marked by BrdU-positive duct cells. Not only were neogenic islets not found, but serial pancreatomy led to an almost complete absence of insulin-positive cells in neogenic areas. Rather, the few islets found in areas of regenerating pancreas appeared to be derived from the area of the pancreas that had not been pancreatectomized [18]. In humans, no evidence for β-cell regeneration was found after partial pancreatectomy [19]. β-cell neogenesis during development requires the transcription factor geurogenin-3, but it appeared to not play a role in β-cell regeneration following partial pancreatectomy [20]. Thus, there does not appear to be support for β-cell regeneration by reactivation of an embryonic program following partial pancreatectomy, and the evidence for any significant β-cell regeneration following partial pancreatectomy is equivocal.

### 3.2. Pancreatic Duct Ligation

Under the hypothesis that β-cell regeneration in adults occurs from ducts, duct ligation has been studied intensively as a model for β-cell regeneration [21]. Most studies, including from us [13], have found small numbers of cells within ducts that express insulin following duct ligation. Multiple studies, including with genetic lineage tracing, have been performed with the goal of demonstrating β-cell neogenesis from duct precursors. While often regarded as a gold standard method, lineage tracing is prone to artifacts, most often arising from leakiness of the promoter used to drive the gene used to mark the duct cell population being studied. The results have varied, with positive [22,23,24] and negative [25,26] studies having been reported. Most recently, a careful lineage-tracing study demonstrated β-cell neogenesis from ducts [27]. However, as with virtually all the previous studies, the efficiency of β-cell formation was low. Overall, definitive evidence for efficient β-cell neogenesis following duct ligation has not been forthcoming.

### 3.3. Inductive Factors

A number of the potential stimuli for β-cell regeneration involve secreted factors. For instance, a corollary to the fact that islets and β-cells arise from embryonic ducts is that there should be inductive factors in the embryonic pancreas that promote islet and β-cell formation. Epidermal growth factor (EGF) has been studied as a factor that can induce expansion of endocrine progenitors in the embryonic and adult pancreas [28,29], but robust increase in β-cell mass has proven elusive. HGF (hepatocyte growth factor) is expressed in both the fetal and adult pancreas and has been claimed to induce the replication of human β-cells [30], but this is controversial [31].

In a more general approach to testing the hypothesis that factors from the fetal pancreas could induce β-cell formation from adult duct cells, we purified non-endocrine epithelial cells from the adult human pancreas, consisting of a population of cells with duct-like properties that did not express insulin, and genetically marked them using a lentiviral vector expressing GFP (green fluorescent protein). Those cells were combined with human fetal pancreatic tissue and transplanted into immunodeficient mice. Following harvest, the existence of cells coexpressing GFP and insulin was determined. There was clear evidence for induction of insulin-positive cells from the duct-like cells under the influence of factors from the co-transplanted human fetal pancreatic tissue. However, the number of insulin-GFP co-positive cells was small, similar to the small number of insulin-positive cells observed in damage models, such as duct ligation [14].

Prolactin, a factor that is at a high level during pregnancy, has been the subject of much investigation as an inducer of β-cell replication during pregnancy [32]. However, while it is clear that there is substantial expansion of β-cell mass in rodents, the evidence for expansion of β-cell mass in humans is weaker than in rodents [33] and if it does occur is likely to be of a much lower magnitude.

Reg is a protein secreted by pancreatic exocrine cells following injury that has been proposed to induce β-cell regeneration [34]. A peptide derived from Reg termed INGAP (islet neogenesis-associated protein) has also been studied as a promoter of β-cell regeneration [35] but has not proven to be robustly active [36].

Overall, no factor from the fetal or adult pancreas has demonstrated robust and reproducible ability to induce β-cell regeneration.

## 4. Dedifferentiation/Redifferentiation

There has been a great deal of interest in the possibility that stressed β-cells, due either to immune attack in type 1 diabetes or lipotoxicity in type 2 diabetes, could dedifferentiate into a state in which β-cell function and insulin expression are decreased, making the dedifferentiated cells resistant to killing by the stressor [37]. If the stressor could be reduced or eliminated, the hope is that dedifferentiated β-cells could redifferentiate to regain β-cell function. For this model to be useful in terms of an approach to the recovery of β-cell function in diabetes, the dedifferentiated cells should remain viable for a considerable period of time and retain the ability to regain normal β-cell function. In type 1 diabetes, the hope is that a dedifferentiated β-cell would lose markers that made it a target for immune attack. In type 2 diabetes, the notion is that a dedifferentiated β-cell would lose the properties that made it a target for lipotoxicity.

The concept of islet cell dedifferentiation received substantial impetus from the Accili lab, which studied β-cell specific deletion of the transcription factor FoxO1 [38]. They found that loss of this factor in β-cells led to loss of insulin from β-cells that had been genetically marked using recombination induced by an Ins2-cre transgene. However, they did not study the fate of the dedifferentiated β-cells in the long term and did not demonstrate the ability of the dedifferentiated cells to regain β-cell function.

In type 2 diabetes, there is evidence for the existence of β-cells that have reduced insulin expression and/or lack the expression of other markers of β-cell function [39,40], which has been taken to supporting the existence of dedifferentiated β-cells [40]. However, it is very difficult to determine whether those cells persist long-term and retain the ability to regain β-cell function. While type 2 diabetes is reversible early in the course of disease, particularly with bariatric surgery, reversibility declines with increasing duration of diabetes [41], suggesting that dedifferentiated β-cells, if they exist in large numbers, have a finite ability to redifferentiate.

In type 1 diabetes, it has been more difficult to demonstrate reversal of disease. Various immunosuppressive regimens have been tried but there has been more success in delaying the progression of autoimmune diabetes than there has been with trying to reverse longstanding disease [42]. Whether that reflects problems with the immunosuppressive regimens or whether there are simply no dedifferentiated β-cells or other β-cell precursors that are primed to undergo β-cell differentiation is unknown.

A prediction of a model in which stably dedifferentiated β-cells remain for long periods in patients with diabetes is that the dedifferentiated cells should remain in the islet, being visible as a substantial number of intra-islet cells lacking insulin. Presumably, these would retain a commitment to the β-cell lineage. In type 1 diabetes, islet morphology is disturbed [43,44]. Cells with low-level insulin expression remain, but those do not appear to be dedifferentiated β-cells [45]. In type 2 diabetes, islet morphology is altered as well [44], and in humans (but not rodents) there is deposition of islet amyloid that appears in place of cells that have apparently been lost [46,47]. If large numbers of dedifferentiated β-cells persist in individuals with longstanding type 2 diabetes, it is unclear where they are.

## 5. β-Cell Neogenesis by Transdifferentiation from Other Islet Cells

There has been substantial interest in the possibility that β-cell neogenesis may occur by transdifferentiation from other islet cells. The first hint that such a pathway may exist came in 1997 from the Teitelman lab, which found that β-cell ablation using the β-cell toxin streptozotocin that acts on cells expressing the glucose transporter GLUT2 led to the appearance of cells co-expressing somatostatin and PDX-1, followed by cells co-expressing somatostatin, PDX-1, and insulin [48]. However, the number of insulin-positive cells generated was low, and no mechanism for the observed transdifferentiation was put forth. In 2010, the Herrera lab and our laboratory published studies demonstrating β-cell neogenesis by transdifferentiation from α-cells [13,49].

The Herrera lab used β-cell ablation, similar to the previous studies from the Teitelman lab, but they increased the efficiency of β-cell ablation with a diphtheria toxin transgenic mouse, as well as lineage tracing to demonstrate more clearly the process of transdifferentiation [49]. However, similar to the studies from the Teitelman lab, the number of β-cell-derived cells that expressed insulin was low and no mechanism was put forth for transdifferentiation.

**Figure 1:**
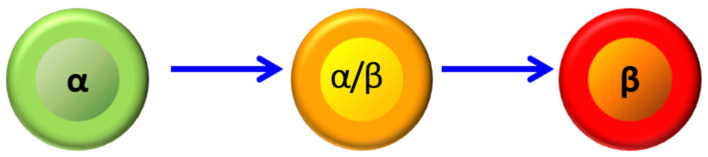
α- to β-cell transdifferentiation. A-cells expressing glucagon (green) transdifferentiate to b-cells expressing insulin (red) through an intermediate co-expressing glucagon and insulin (yellow). Scale bar 10 μm.

Our studies on transdifferentiation began with an attempt to reproduce the studies that used duct ligation to stimulate β-cell neogenesis. Similar to others, we found an increase in insulin-positive cells in ducts [13], but also similar to others, those cells were uncommon. To study the origin of the intra-ductal insulin-positive cells, we ablated preexisting β-cells using alloxan, which acts similarly to streptozotocin, simultaneously with duct ligation. Surprisingly, this led to a massive increase in the number of cells expressing insulin. However, those cells did not form within ducts. Rather, there was a massive increase in cells co-expressing insulin and glucagon. Those cells gradually lost glucagon expression and became functional β-cells (Figure 1) [13]. Pancreatitis appeared to play an important role, as caerulein-induced pancreatitis also led to massive α- to β-cell transdifferentiation [43]. We hypothesized that an exocrine enzyme released by pancreatitis was important and tested the hypothesis that trypsin and its target, protease-activated receptor 2 (PAR2), was playing a role. PAR2 was found to be highly expressed in islets, including β-cells. Studies with the PAR2 agonist drug 2-Furoyl-LIGRLO-amide trifluoroacetate and the PAR2 knockout mouse demonstrated that PAR2 was required for α- to β-cell transdifferentiation [50]. Interestingly, PAR2 appeared to play a much broader role in regeneration, being important in hepatic regeneration following carbon tetrachloride administration and digit regeneration following amputation [50]. The requirement for β-cell ablation was explained by the finding that PAR2 activation was suppressed by insulin [51]. Of note for pharmacological relevance, glucagon-insulin double-positive cells, an intermediate in transdifferentiation, could be induced by a PAR2 agonist in combination with the inhibitor of insulin secretion, diazoxide.

## 6. Conclusions

Many routes to the restoration of β-cell function in diabetes have been proposed [52]. However, many interventions that have been claimed to increase β-cell mass lack a connection to a physiologically relevant pathway. It is hard to understand why a pathway that does not respond to a physiologically relevant situation should even exist.

Early in the course of disease, the evidence supports that a substantial number of preexisting β-cells persist, so restoration of function may be accomplished by eliminating the stimulus for β-cell loss, which in the case of type 1 diabetes would be β-cell autoimmunity and in the case of type 2 diabetes would be lipotoxic stress. As the duration of diabetes increases, the best evidence supports that β-cell loss due to death occurs, so once that has progressed to the point of clinical diabetes, reversal would require β-cell neogenesis. To date, no pathway to β-cell neogenesis has been definitively proven, particularly in humans.

The evidence that β-cell replication can occur in adult mammals is weak, with no studies demonstrating a large increase in β-cell mass. Age-related obstacles to replication exist that may be insurmountable but it is possible that β-cell replication could be applicable in patients with type 1 diabetes, who most commonly develop disease in childhood, when replicative potential may remain. Neogenesis from duct cells in adults appears to be extremely limited. While there does appear to be some capacity for this to occur, no studies have demonstrated that it can result in a large increase in β-cell mass. Regardless of the absence of good evidence for the generation of large numbers of β-cells by β-cell replication or neogenesis from ducts, the evidence for low-level occurrence of those processes is suggestive, providing impetus for efforts to increase their efficiency.

To date, the best evidence for robust β-cell regeneration comes from transdifferentiation from other islet cells. However, even there, only limited evidence exists that this process can occur in humans [43,50,52]. The combination of a PAR2 agonist plus diazoxide was tested in vitro, but in vivo translation, while theoretically possible, would be challenging in type 2 diabetes. For type 1 diabetes, PAR2 activation would need to be coupled with avoidance of recurrent autoimmunity, but progress is being made along those lines [53].

Efforts to demonstrate meaningful β-cell regeneration in humans would benefit from greater emphasis on ruling out sources of artifact and false positive results. The devastating impact of diabetes has promoted a grasping at straws in the hope of developing a treatment or even cure, but this has led to wasted time and effort on many blind alleys. The field of β-cell regeneration would benefit from more attention to the principle of falsifiability described by Karl Popper [54] but there does appear to be promising routes to regeneration that could eventually be translated to humans.
